# Randomized, placebo-controlled, double-blind, pilot trial to investigate safety and efficacy of Cerebrolysin in patients with aneurysmal subarachnoid hemorrhage

**DOI:** 10.1186/s12883-020-01908-9

**Published:** 2020-11-03

**Authors:** Peter Y. M. Woo, Joanna W. K. Ho, Natalie M. W. Ko, Ronald P. T. Li, Leo Jian, Alberto C. H. Chu, Marco C. L. Kwan, Yung Chan, Alain K. S. Wong, Hoi-Tung Wong, Kwong-Yau Chan, John C. K. Kwok

**Affiliations:** Department of Neurosurgery, Kwong Wah Hospital, Room CS11-01, 11th Floor, 25 Waterloo Road, Yaumatei, Hong Kong, China

**Keywords:** Aneurysmal subarachnoid hemorrhage, Neuroprotection, Delayed cerebral ischemia

## Abstract

**Background:**

There are limited neuroprotective treatment options for patients with aneurysmal subarachnoid hemorrhage (SAH). Cerebrolysin, a brain-specific proposed pleiotropic neuroprotective agent, has been suggested to improve global functional outcomes in ischemic stroke. We investigated the efficacy, safety and feasibility of administering Cerebrolysin for SAH patients.

**Methods:**

This was a prospective, randomized, double-blind, placebo-controlled, single-center, parallel-group pilot study. Fifty patients received either daily Cerebrolysin (30 ml/day) or a placebo (saline) for 14 days (25 patients per study group). The primary endpoint was a favorable Extended Glasgow Outcome Scale (GOSE) of 5 to 8 (moderate disability to good recovery) at six-months. Secondary endpoints included the modified Ranking Scale (mRS), the Montreal Cognitive Assessment (MOCA) score, occurrence of adverse effects and the occurrence of delayed cerebral ischemia (DCI).

**Results:**

No severe adverse effects or mortality attributable to Cerebrolysin were observed. No significant difference was detected in the proportion of patients with favorable six-month GOSE in either study group (odds ratio (OR): 1.49; 95% confidence interval (CI): 0.43–5.17). Secondary functional outcome measures for favorable six-month recovery i.e. a mRS of 0 to 3 (OR: 3.45; 95% CI 0.79–15.01) were comparable for both groups. Similarly, there was no difference in MOCA neurocognitive performance (*p*-value: 0.75) and in the incidence of DCI (OR: 0.85 95% CI: 0.28–2.59).

**Conclusions:**

Use of Cerebrolysin in addition to standard-of-care management of aneurysmal SAH is safe, well tolerated and feasible. However, the neutral results of this trial suggest that it does not improve the six-month global functional performance of patients.

**Clinical trial registration:**

Name of Registry: ClinicalTrials.gov

Trial Registration Number: NCT01787123.

Date of Registration: 8th February 2013.

## Background

Aneurysmal subarachnoid hemorrhage (SAH) accounts for less than 5% of all stroke, but since patients are generally younger and the extent of brain insult often more severe, it is disproportionately responsible for 27% of all stroke-related potential life years lost before the age of 65 [1]. Advances in intracranial aneurysm treatment and neurocritical care have substantially improved outcomes, but delayed cerebral ischemia (DCI) continues to be a management challenge [2–4]. DCI is a major determinant of mortality, accounting for up to 50% of all SAH-related deaths, and morbidity in patients that survive the initial hemorrhage [5, 6]. It occurs in 20–40% of patients, but in spite of its recognition as an important complication of SAH, its exact pathophysiology remains to be elucidated [7, 8]. Since it usually occurs between four and 10 days after SAH, there exists a potential therapeutic window that has been subject to substantial neuroprotective agent research [5]. Clinical trials investigating endothelin receptor antagonists such as clazosentan, lipid peroxidation inhibitors such as tirilazad, aspirin, statins, magnesium, hypertensive therapy, and transluminal balloon angioplasty have all been unsuccessful [9–16]. The only consistently proven neuroprotective treatment for SAH that reduces poor clinical outcomes is nimodipine, a L-type calcium channel blocker that was discovered more than three decades ago [2, 17].

Cerebrolysin (EVER Neuro Pharma GmbH, Unterach, Austria) is an intravenously administered preparation of low-molecular weight neuropeptides of less than 10 kDa (80%) and free amino acids (20%) derived from porcine brain tissue. It is a brain-specific pleiotropic agent that is proposed to target multiple ischemic pathophysiological events due to the combined action of its constituent neurotrophic factors [18–29]. Its neuroprotective properties have been demonstrated in both in vitro and murine ischemic stroke models. They include anti-apoptosis, mitigating glutamate excitotoxicity, reducing free oxygen radical concentrations, microglial activation and neuro-inflammatory response modulation [19–23, 25–27, 30]. Cerebrolysin has also been shown to enhance neuroplasticity by synaptic remodeling and promoting neurogenesis in the peri-infarct zone [18, 20, 24, 29, 31].

Randomized placebo-controlled clinical trials (RCT) evaluating the role of Cerebrolysin in acute ischemic stroke patients have demonstrated improved 90-day functional outcomes with regard to motor performance, the National Institutes of Health Stroke Scale (NIHSS), the modified Rankin Scale (mRS) and the Barthel Index (BI) [32–37]. Although several meta-analyses have been performed, the role of Cerebrolysin in acute ischemic stroke has yet to be defined [38–42]. A recent study, analyzing nine RCTs, revealed encouraging results by observing improved three-month mRS and NIHSS outcomes when early treatment was initiated within 72 h of stroke [40].

In contrast, no RCT has explored the role of Cerebrolysin in aneurysmal SAH where the pathophysiological mechanisms are distinct from ischemic stroke. Especially with respect to DCI, an opportunity exists for candidate neuroprotective agents to be administered before it develops. Only one 10-year retrospective study was previously performed which observed significantly reduced three-month mortality rates among severe SAH patients that received Cerebrolysin and those that underwent microsurgical aneurysm clipping [43]. Therefore, we investigated the potential benefits of Cerebrolysin among aneurysmal SAH patients by conducting a randomized, placebo-controlled, double-blind pilot study.

## Methods

This was an investigator-initiated, single-center randomized, placebo-controlled, double-blind, 1:1 parallel-group phase IIa pilot trial that investigated the effects of Cerebrolysin for consecutive adult patients diagnosed with aneurysmal SAH from 1 February 2014 to 30 June 2018. Clinical research ethics committee approval was obtained (Institutional Review Board number: KW/FR-13-006 (61–04)). The trial was registered with ClinicalTrials.gov (NCT01787123) and was conducted according to the Declaration of Helsinki and Good Clinical Practice. All subjects or their legal representatives provided written informed consent.

### Inclusion and exclusion criteria

Patients that fulfilled the study criteria were enrolled by the study investigators. Inclusion criteria were: age 18 to 80 years-old, of Chinese descent with a radiological diagnosis of aneurysmal SAH and where randomization could be performed within 96 h of ictus. Exclusion criteria were: unsalveagble severe brain insult upon presentation (when death was anticipated within 48 h of admission or when there were post-resuscitation signs of central or uncal herniation); pre-existing neurological or psychiatric disorders, including stroke, epilepsy and dementia; a pre-SAH disability (i.e. a mRS of > 2); major cardiac, pulmonary, hepatic and renal disease (i.e. creatinine concentration of > 200 μmol/L); pre-existing terminal medical illness with a life-expectancy of less than a year; an existing diagnosis of DCI or vasospasm; an active history of alcohol or illicit drug dependency or a previous history of Cerebrolysin exposure. Patients with any contraindication for Cerebrolysin administration, including pregnancy, lactation and allergies to its components were also excluded.

### Subject management, randomization and blinding

After establishing the diagnosis of a ruptured intracranial aneurysm by either computed (CT) tomography angiography or catheter angiography, patients were randomly assigned to receive either intravenous Cerebrolysin (intervention group) or normal saline infusions (placebo group). In prospective studies focused on ischemic stroke, Cerebrolysin dosages varied from 10 to 50 ml per day [32, 34, 37, 44, 45]. According to two phase III RCTs, a daily dose of 30 ml Cerebrolysin was administered for a duration of 10 to 21 days [32, 44]. It was decided to adopt a similar daily dose for 14 days in order to cover the period where DCI most frequently occurs after SAH [46]. Subjects in the intervention group received 30 ml of IV Cerebrolysin per day for 14 days. Eight-hourly 30-min IV infusions consisting of 10 ml of Cerebrolysin diluted with saline to a total volume of 100 ml was given. Subjects in the control group received 100 ml of saline every 8 h for the same period. Allocation was performed according to a predefined block randomization plan generated by the Statistical Package for the Social Sciences version 22.0 (SPSS Inc., Chicago, IL, US). A block size of 10 was used and group allocation within each block was conducted with a 1:1 ratio. Assignment instructions were sealed in envelopes and opened after subject recruitment. Patients and study outcome assessors were blinded to group identity while clinicians directly involved in their medical management were aware. Since Cerebrolysin carries a yellow tint, infusion bags were wrapped in opaque plastic and amber-coloured IV tubing were utilized for both study groups in order to mask the infusate administered.

All recruited subjects were treated according to the latest 2012 American Heart Association (AHA)/ American Stroke Association (ASA) Guidelines for the Management of Aneurysmal SAH [47]. In the initial phase all patients were treated at the neurocritical care unit with adherence to Class I and IIa recommendations for the management of DCI: four-hourly doses of Nimodipine 60 mg was administered for 21-days, euvolemia was maintained, induction of hypertension was performed if DCI was diagnosed and intra-arterial vasodilator therapy was performed in patients with symptomatic cerebral vasospasm unresponsive to hypertensive therapy [47]. When considered fit for post-stroke rehabilitation, all subjects were enrolled into a minimum two-week standardized early inpatient mobilisation physiotherapy program and occupational therapy for basic activities of daily living training.

### Data collection and study endpoint assessment

Data from clinical records, operation notes, medication dispensing records, laboratory and radiological investigations were collected by an independent neurosurgeon, with 5 years of specialist experience, without knowledge of the subject’s group assignment. Clinical severity of SAH was classified according to the modified World Federation of Neurosurgical Societies (WFNS) grading scale. This categorization is regarded as a core data element for SAH trials according to the National Institutes of Health (NIH)/ National Institute of Neurological Disorders and Stroke (NINDS) Common Data Elements Project (CDE) [48, 49]. Good-grade WFNS was defined as grade I or II, i.e. patients presenting with a GCS of 14 or 15. The Acute Physiology and Chronic Health Evaluation (APACHE II) score, an independent predictor for in-hospital mortality for SAH patients, was also calculated [50]. The Charlson Comorbidity Index (CCI), a validated prognostic system for ischemic stroke patients, comprising of a weighted score of 17 comorbidities based on the International Classification of Diseases, Ninth Revision, was determined [51, 52]. The degree of SAH on the first CT brain scan was evaluated according to the modified Fisher’s grading and Hijdra scoring systems [53, 54]. The Hijdra system consists of a semiquantitative assessment of the amount of blood identified in 14 regions of interest with a score of > 22 being an independent predictor for poor mRS functional outcome at six-months [55].

Global functional outcome was evaluated by an independent assessor, a registered nurse, at 30 days, 3 months and 6 months after ictus using the following instruments: the Extended Glasgow Outcome Scale (GOSE), mRS and BI. The primary endpoint was favourable GOSE performance, defined as grades 5 to 8 (moderate disability to good recovery) at 6 months after ictus. Multi-dimensional secondary study endpoints were also assessed: mRS, BI, neurocognitive function and quality of life at 30 days, three- and six-months. In particular, mRS was considered as a highly recommended outcome measure for disability according to the CDE Project working group and favourable recovery was defined as a score of 0–3 (asymptomatic to moderate disability) [49, 56]. A BI cut-off score of > 75 was also utilized to define a favourable outcome [57]. Neurocognitive performance was evaluated by the Montreal Cognitive Assessment (MOCA), another highly recommended CDE Project outcome metric, and the Neurobehavioral Cognitive State Examination (NCSE) [49, 56]. Quality of life (QoL) was appraised by adopting the Chinese version of the 36-item Short Form Health Survey questionnaire (SF-36) and the Stroke-specific QOL Scale (SS-QOL). The occurrence of DCI, cerebral vasospasm and radiological evidence of cerebral infarction were determined by an independent neurosurgeon and neuro-radiologist. DCI was defined as a decrease in GCS of > 2 points or the development of focal neurological deficit for at least 1 h not related to post-treatment complications, rebleeding, hydrocephalus, infection, electrolyte/ metabolic disturbances or seizures according to Vergrouwen et al. and recommended by a recent systematic review of standardized SAH outcomes [5, 58]. According to our institution’s management protocol, seizures were detected by performing bedside electroencephalography (EEG) for all patients with delayed clinical deterioration or if they had a history of seizures on presentation. Cerebral vasospasm was defined as angiographically detectable moderate-to-severe arterial narrowing not attributable to atherosclerosis, catheter-induced spasm or vessel hypoplasia. A transcranial Doppler ultrasound reading of the middle cerebral artery (MCA) with a mean velocity of > 120 cm/sec or a Lindegaard ratio, MCA: internal carotid artery (ICA) mean velocity, of > 3 was considered indicative of cerebral vasospasm [6]. SAH-related cerebral infarction was defined as demonstrable CT or MRI evidence within 6 weeks of ictus, that was absent on scans performed 24 to 48 h after aneurysm occlusion and was not judged to be a complication of neurosurgical intervention [5]. Finally, 30-day mortality, three- and six-month mortality were also recorded.

### Safety evaluation

Cerebrolysin-related severe adverse effects (SAEs) are rare. They are defined as hypersensitivity reactions such as anaphylactic shock, seizures and acute renal failure [59]. Other AEs are generally infrequent, transient and mild: agitation, headache, vertigo, gastrointestinal symptoms such dyspepsia, diarrhoea, constipation, nausea and vomiting [59].

Patients assigned to receive Cerebrolysin were monitored by the treating clinician for any changes in vital signs as well as in their general physical and neurological examinations. Laboratory tests were evaluated for abnormalities attributable to Cerebrolysin. If SAEs occurred the decision for premature trial termination was made by the study investigators.

### Statistical analysis

Since the treatment effects of Cerebrolysin in aneurysmal SAH are unknown, to determine the sample size for this pilot study, trials that focused on ischemic stroke were used as a reference. The largest phase III trial for Cerebrolysin in ischemic stroke determined that a sample size of 990 subjects, with an α-level of 0.025 (one-sided) and 90% power, was required to detect treatment superiority over standard care alone for functional performance at 3 months [44]. By adopting a Bayesian decision-theoretic approach, it was proposed that phase II trials should have a sample size approximately 0.03 times that of a subsequent phase III study, which in this case would be 30 (= 990 × 0.03) subjects in total [60]. However, it was ultimately decided to increase the sample size to 50 subjects since Sim et al. advocated that this number was the absolute minimum required to decide on whether to proceed with a main RCT with a 5% two-tailed α-level and 80% power [61]. All analyses were performed on a modified intention-to-treat basis where the last available observed outcome measure was carried forward to handle missing data. In addition to assessing dichotomized six-month GOSE and mRS outcomes as either favourable or unfavourable, their ordinal character lends itself to proportional odds analysis. This approach was described by Weir et al. to have superior efficiency compared to the conventional binary analysis and was therefore also utilized in this study [62]. Prespecified subgroup analyses were performed for age (> 65 or < 65 years), pre-existing hypertension, modified WFNS grade (good-grade: I, i.e. an admitting GCS of 15 or II, i.e. GCS of 14 versus poor-grade: III, i.e. GCS 13; IV, i.e. GCS 7–12 or V, i.e. GCS 3–6), modified Fisher grading (I, II or III, IV), aneurysm location (anterior or posterior circulation) and treatment modality (endovascular therapy or microsurgical clipping). The reporting of this study was in accordance with the recommendations outlined in the Consolidated Standards of Reporting Trials (CONSORT) statement [63]. Statistical tests included logistic regression, the chi-squared test, the independent-samples t-test, the Wilcoxin Mann-Whitney U-test and proportional odds analysis. An α-level of 0.05 was used to define statistical significance. Tests were performed by either using Statistical Package for the Social Sciences software version 20.0 (SPSS Inc., Chicago, Illinois, USA) or R version 3.3.2 (R Foundation for Statistical Computing, Vienna, Austria).

## Results

**Fig. 1:**
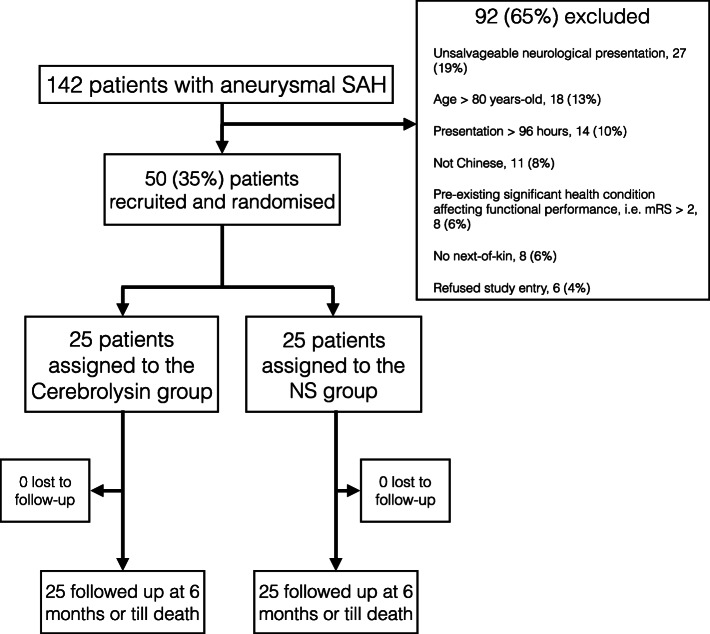
Trial subject profile

A total of 142 patients were diagnosed with aneurysmal SAH between 1 February 2014 and 26 June 2018. The most frequent reason for exclusion was unsalvageable neurological presentation (19%, 27/142) followed by advanced age (13%, 18/142) and delayed neurosurgical treatment beyond 96 h after ictus (10%, 14/142) (Fig. 1). Among them 50 patients (35%, 50/142) were enrolled into the pilot study with 25 assigned to the intervention or control groups respectively. All intervention group subjects completed 14 days of Cerebrolysin administration. There were no premature trial terminations, no protocol violations and follow-up assessements were complete for all subjects. The baseline patient-, disease and treament- characteristics between the two groups were similar (Table 1). The mean time from ictus to Cerebrolysin or placebo infusion was 29 + 15 h. The mean age was 53 years + 10 (range: 34–78 years) with a male: female ratio of 1:2. Patients from both groups had relatively few co-morbidities with comparable CCIs. The median modified WFNS grade was II and the majority of patients presented with good-grade SAH (30, 60%) with a mean APACHE II score of 10 + 5. Regarding the amount of SAH on the initial CT scan, the median modified Fisher grade was III and the mean Hijdra score was 17 + 7. Most intracranial aneurysms were located at the ICA (42%, 21/50) followed by the anterior communicating artery (AComA) (24%, 12/50) and the MCA (20%, 10/50). The majority of aneurysms were treated by endovascular therapy (70%, 35/50) and the remaining were clipped. No SAEs or mortality attributable to Cerebrolysin were observed. Drug administration did not interfere with standard-of-care management.

**Table 1:** Baseline Characteristics of Patients in the Cerebrolysin and Saline Groups

	Cerebrolysin *n* = 25	Saline *n* = 25	*P*-value
**Patient Factors**			
Age, mean + SD (range), years	52 + 9 (31–65)	54 + 11 (37–70)	0.72
> 65 years	2	4	0.67
Female	16	18	0.76
Smoker	12	9	0.39
Pre-existing hypertension	11	5	0.07
Baseline MAP, mean + SD, mmHg	127 + 28	122 + 22	0.45
Median CCI	1	1	1.00
**Disease Factors**			
Modified WFNS			0.79
I	11	14	
II	4	1	
III	1	3	
IV	8	6	
V	1	1	
APACHE II score, mean + SD	11 + 5	9 + 5	0.14
Modified Fisher CT Grade			0.07
I	3	7	
II	3	5	
III	12	6	
IV	7	7	
Hijdra score, mean + SD	17 + 6	16 + 7	0.64
Hijdra score > 22	6	8	0.53
Hydrocephalus	11	11	1.00
Intraventricular hemorrhage	10	10	1.00
Intracerebral hemorrhage	6	6	1.00
Rebleeding	0	1	1.00
Aneurysm location			0.53
ICA	10	11	
ACA/ AComA	5	9	
MCA	7	3	
PC	3	2	
**Treatment Factors**			
Aneurysm treatment			
Endovascular therapy	16	19	0.11
Clipping	9	6	0.36

N.B. *MAP* mean arterial pressure, *CCI* Charlson comorbidity index, *WFNS* World Federation of Neurosurgical Societies, *APACHE* Acute Physiology and Chronic Health Evaluation, *IQR* interquartile range, *CT* computed tomography, *ICA* internal carotid artery, *ACA* anterior cerebral artery, *AComA* anterior communicating artery, *MCA* middle cerebral artery, *PC* posterior circulation

**Fig. 2:**
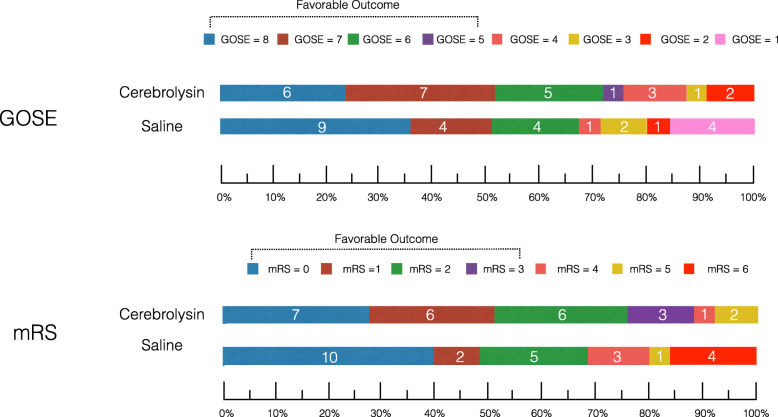
Distribution of six-month GOSE and mRS global functional outcomes for Cerebrolysin and saline group subjects

Analyses were performed according to the originally assigned study groups (Fig. 2 and Table 2). There was no significant difference in favourable six-month GOSE outcome among subjects that received Cerebrolysin (76%; 19/25) compared to those that received saline (68%; 17/25) (OR 1.49; 95% CI 0.43–5.17) (Fig. 2 and Table 2). Although a higher proportion of Cerebrolysin subjects had favourable six-month mRS scores (88%; 22/25) compared to the saline group (68%; 17/25) the difference was not significant (OR: 3.45; 95% CI 0.79–15.01). Similar observations were made with regard to the number of subjects in each group with a six-month BI > 75 (OR: 4.47; 95% CI: 0.83–24.19). Adopting a proportional odds model, GOSE outcomes were subgrouped into 1–4 (death/ vegetative state/ severe disability), 5–6 (moderate disability) and 7–8 (good recovery). mRS was subgrouped into 0–2 (asymptomatic to slight disability), 3–4 (moderate disability) and 5–6 (severe disability/ death). Ordinal analysis of six-month GOSE (*p*-value: 0.80) and mRS (p-value: 0.76) also revealed no significant difference in outcome between Cerebrolysin and saline group subjects.

**Table 2:** A Comparison of Outcomes in the Cerebrolysin and Saline Groups

	Cerebrolysin *n* = 25	Normal Saline *n* = 25	OR (95% CI)
**Global Functional Outcome at 6 months**			
GOSE, median (IQR)	7 (5–8)	7 (3–8)	NS
GOSE, favorable outcome, i.e. 5–8	19	17	NS
mRS, median (IQR)	1 (0–3)	2 (0–4)	NS
mRS, favorable outcome, i.e. 0–3	22	17	NS
BI, mean + SD	91 + 28	90 + 24	NS
BI > 75	23	18	NS
**Global Functional Outcome at 3 months**			NS
GOSE, median (IQR)	5 (4–6)	6 (3–7)	
GOSE, favorable outcome, i.e. 5–8	15	16	NS
mRS, median (IQR)	2 (2–4)	2 (2–5)	NS
mRS, favorable outcome, i.e. 0–3	18	17	NS
**Mortality**			
**6-month**	0	4	0.46 (0.33–0.63)
**3-month**	0	4	0.46 (0.33–0.63)
**30-day**	0	3	NS
**Delayed Cerebral Ischemia**	11	12	NS
**Cerebral Vasospasm**	3	7	NS
**Cerebral infarction**	14	15	NS
**Neurocognitive Performance at 6 months**			
MOCA, mean + SD	21 + 9	21 + 8	NS
NCSE, within average range			
Orientation	20	18	NS
Attention	21	18	NS
Language comprehension	18	16	NS
Repetition	15	10	NS
Naming	11	3	4.71 (1.10–20.00)
Constructional ability	19	15	NS
Memory	12	9	NS
Calculation	19	17	NS
Reasoning	18	10	2.83 (1.01–9.61)
Judgment	19	18	NS
**Quality of Life at 6 months**			
SF-36®			
Physical health score, mean + SD	77 + 23	74 + 26	NS
Mental health score, mean + SD	69 + 25	69 + 21	NS
SS-QoL, mean + SD	4.5 + 0.7	4.5 + 0.6	NS
Physical subscore, mean + SD	4.6 + 0.8	4.5 + 0.7	NS
Psychosocial subscore, mean + SD	4.2 + 0.9	4.4 + 0.6	NS
**Adverse Effects**	0	0	NS
**Length of Hospital Stay, mean** **+** **SD**	33 + 15	39 + 32	NS

*OR* odds ratio, *CI* confidence interval, *mRS* modified Rankin score, *GOSE* extended Glasgow outcome score, *IQR* interquartile range, *SD* standard deviation, *BI* modified Barthel index, *MOCA* Montreal cognitive assessment, *NCSE* Neurobehavioral cognitive state examination, *SF-36®* Short-form 36 Health Survey, *SS-QoL* stroke-specific quality of life, *NS* not significant

A higher incidence in three- and six-month mortality was observed in saline group subjects than in the Cerebrolysin group. 16% (4/25) of saline group subjects died at these times points while all Cerebrolysin group subjects survived (ORs 0.46; 95% CI 0.33–0.63). The cause of death for three of these patients (75%, 3/4) was due to medically-refractory intracranial hypertension arising from DCI-induced cerebral edema and the remaining patient died from a chest infection. A review of the incidence of inpatient SAH-related complications such as cardiac failure, acute myocardial infarction, renal failure, chest infection, septic shock, pulmonary embolism or gastrointestinal bleeding revealed comparable frequencies of occurence between the two study groups (*p*-values > 0.05). No association was detected between three- and six-month mortality and poor-grade SAH subjects (*p*-value: 0.57) or for those that underwent microsurgical clipping (p-value: 0.81).

Overall, DCI was detected in 46% (23/50) of subjects and the proportion of patients in each group were similar (OR 0.85; 95% CI 0.28–2.59). Upon serial imaging, cerebral infarction was detected in the majority of patients (58%, 29/50), but there was no significant difference in incidence between the study groups (OR 0.85; 95% CI 0.28–2.61). Cerebral vasospasm was diagnosed in 20% (10/50) of all patients. More than twice as many saline group patients (28%, 7/25) had vasospasm than those in the Cerebrolysin group (12%, 3/25), but this also did not reach statistical significance (OR 0.35; 95% CI 0.08–1.55).

Six-month neurocognitive and QoL assessments were feasible in 80% (40/50) of patients since the remaining were non-commuicable (Table 2). The mean MOCA scores were similar, 21 + 9 in the Cerebrolysin group and 21 + 8 in the saline group (independent-samples t-test *p*-value: 0.75). Most of the assessed domains for the NCSE were comparable, but subjects in the Cerebrolysin group performed notably better in naming (OR 4.71; 95% CI 1.10–20.00) and in reasoning (OR 2.83; 95% CI 1.01–9.61). For six-month QoL assessments, mean SF-36 physical and mental scores as well as the mean physican and psychosocial subscores for SS-QoL were similar for both groups (independent-samples t-test *p*-values > 0.05).

**Fig. 3:**
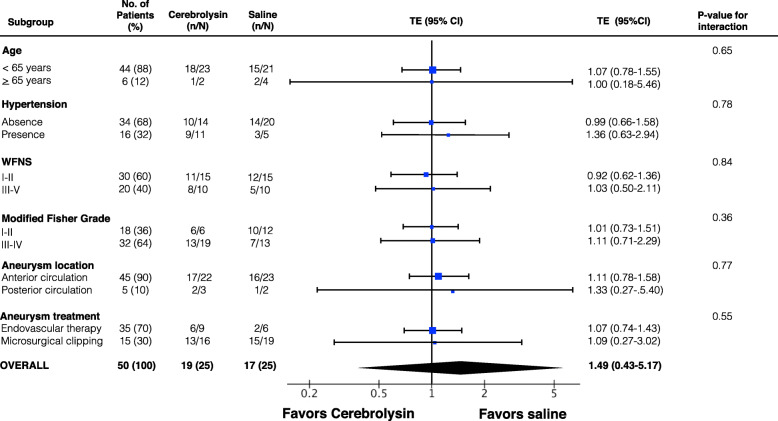
Forest plot. A priori subgroup analysis for favorable GOSE (5 to 8) at 6 months. n, number with favorable GOSE in each subgroup. N, total number randomized in each subgroup. TE, treatment effect. WFNS, World Federation of Neurosurgical Societies

Predefined subgroup analyses for favourable six-month GOSE by age (cut-off at 65 years), pre-existing hypertension, WFNS grade, modified Fisher grading, aneurysm location and treatment modality also did not reveal significant superiority for Cerebrolysin (Fig. 3).

## Discussion

To our knowledge this is the first RCT to investigate the role of Cerebrolysin for aneurysmal SAH. In addition, no previous Cerebrolysin stroke study has reported its effect on six-month functional outcomes despite its asserted neurorestorative potential during the recovery phase [32, 35, 36]. Cerebrolysin administration was well tolerated and feasible for the acute treatment of aneurysmal SAH patients. The drug showed a satisfactory safety profile with no increased incidence of SAEs or mortality. There was also no interference with standard-of-care treatment according to AHA/ASA guidelines and administration within 96 h of ictus was achievable. However, the results of this trial were neutral. Cerebrolysin group subjects did not have improved global functional performance at either three- or six-months. Regardless of the statistical analysis approach adopted, i.e. fixed dichotomy or proportional odds modeling, there was no significant difference in GOSE or mRS outcomes between the study groups. It was believed that the latter strategy could reduce the loss of information about outcome and increase statistical power as demonstrated by previous TBI and stroke trials [64, 65]. But the lack of efficiency gains with proportional odds modeling observed in this study supports the similarly neutral findings of a reanalysis of pooled data from seven SAH trials that also compared these two statistical approaches [66].

A major reason for the neutral results of SAH studies investigating neuroprotective agents was the lack of consistency in outcome measures [58]. To address this, the NIH/ NINDS Common Data Elements Project for SAH was initiated in an attempt to harmonize and standardize data collected for clinical research [49]. A review of more than 50 outcome measures considered relevant for SAH was performed by an international, multidisciplinary working group consisting of experts in the fields of neurology, neurosurgery and neurorehabilitation. Although no core outcome measures were identified, two assessments were highly recommended, namely the mRS and the MOCA [49, 56]. Both of these data elements were documented in the present study, but no discernible differences were observed between the study groups (Table 2). Since the present trial was initiated before the CDE Project consensus was published, we continued to adopt GOSE as the primary outcome not only because of its utility in previous phase III SAH trials, but also due to its superior sensitivity to detect changes in outcome compared to conventional GOS [10, 14, 62].

Three- and six-month mortality rates were significantly lower among Cerebrolysin patients and corroborates the findings of Park et al. in their 10-year retrospective study of 462 SAH patients [43]. In that study the three-month mortality rates of patients that received Cerebrolysin were 9% for those with poor-grade SAH and 7% for those that underwent aneurysm clipping [43]. These figures were considerably lower than the recorded respective mortality rates of 25 and 19% among those that received only standard-of-care treatment [43]. Among its various mechanisms of action Park et al. postulated that Cerebrolysin also ameliorated the deletrious effects of cerebral edema [43]. Murine intracerebral hemorrhage models exposed to Cerebrolysin revealed significant reductions in pro-inflammatory markers such as IL-β, IL-6 and TNF-α as well as aquaporin-4, an important mediator for hematoma-induced vasogenic edema [67]. Electron microscopy also demonstrated reduced astrocytic cytotoxic edema and secondary brain injury changes with Cerebrolysin [67]. In the present study, the cause of death for 75% (3/4) of control group subjects was DCI which would have resulted in cytotoxic cerebral edema. However, since the majority of subjects in the present study had milder SAH and underwent endovascular treatment, the trial sample size was not adequately powered to assess this endpoint and our findings on reduced mortality could have been coincidental.

Another frequently proposed reason for the neutral results of numerous clinical SAH trials is that candidate neuroprotective agents often addressed a single process in the neuronal ischemic cascade [68, 69]. In contrast, Cerebrolysin targets multiple pathophysiological events [18–29]. The exact mechanisms of action are unknown, but preclinical studies have suggested that Cerebrolysin’s neuroprotective properties are mediated via enhanced anti-apoptosis, regulation of glutatmate excitotoxicity and neuroinflammation [21, 23, 25, 27, 28, 30]. Neuroplasticity reflected by intensified synaptic remodeling and transmission have also been observed in the murine neocortex and hippocampus [29, 31]. Cerebrolysin-induced neural progenitor cell proliferation at the subventricular zone and neurogenesis at the peri-infarct zone by activation of the Sonic hedgehog and PI3K/Akt signaling pathways have been observed [18, 20, 24]. Finally, several ischemic stroke model animal studies have also detected significant improvements in sensorimotor and cognitive recovery with Cerebrolysin [18, 19, 22, 29].

To date five meta-analyses of RCTs investigating the efficacy of Cerebrolysin in acute ischemic stroke have been performed [38–42]. Three, evaluating aggregate data from up to seven trials involving 1501 to 1779 patients, concluded that the medication did not demonstrate any clinical benefit [38, 41, 42]. In particular, a Cochrane Review did not find Cerebrolysin to be useful, but several trials were excluded from the analysis since treatment initiation exceeded the review protocol’s prespecified 48-h stroke-onset window [38]. A recent larger meta-analysis of nine RCTs involving 1879 patients, drew contrasting conclusions observing that Cerebrolysin resulted in improved three-month mRS outcomes in moderate to severe stroke patients and significant improvements in NIHSS scores with the number needed to treat being 7.7 [40]. In addition to global functional outcomes, other RCTs also documented Cerebrolysin’s therapeutic potential in enhancing neurocognitive performance, reducing infarct volumes as well as increasing neuroplasticity signal changes of the motor corticospinal tract by diffusion tensor imaging [35, 37, 45, 70, 71].

Compared to ischemic stroke, where focal cerebral infarction develops within hours, SAH is a global hemorrhagic brain insult that can result in DCI in 20–40% of patients [5, 6]. We hypothesized that the early administration of Cerebrolysin before DCI onset would have a prophylactic or ameliorating effect that could translate to improved functional outcomes. But improved outcomes were not detected in the present study and was likely due to its limited sample size. A condition sharing comparable characteristics to aneurysmal SAH in terms of the extent of insult and DCI is traumatic brain injury (TBI). Post-traumatic delayed ischemia can develop five to 7 days after severe TBI in 19–68% of patients [72]. In support of our postulation, a retrospective case-matched historical cohort study of 129 severe TBI patients documented significantly improved six-month favorable GOSE functional outcomes for patients that received Cerebrolysin and lower mortality [73]. A recent RCT of moderate-to-severe TBI patients also revealed that Cerebrolysin, given within 6 h of injury, resulted in improved three-month neuro-psychological executive functional performance [74]..

The long time-windows incurred from stroke onset to neuroprotective agent administration could also explain why several clinical trials have failed to deliver positive results [68, 75]. For this study there could have been an underestimation of the impact of early brain injury (EBI) immediately following SAH. The concept of EBI has gained precedence as a therapeutic target in recent years [76]. SAH-induced EBI triggers blood-brain barrier dysfunction, inflammation, apopotosis, oxidative stress, cortical spreading depression and excitotoxicity, that can be equally or more predictive of morbidity than DCI [76, 77]. Investigators of neuroprotective stroke trials have increasingly advocated the importance of ultra-early intervention, i.e. within the first four to 6 h of symptom onset [68, 69, 75, 76]. In support of earlier intervention for SAH neuroprotection, ischemic stroke and TBI trials, demonstrated significant improvements in three-month neurological outcomes when Cerebrolysin was administered within 6 h after hospital admission [34, 37, 74]. In the present study the mean duration for initiation of Cerebrolysin was 29 h and therefore we propose that future trials should consider implementing an earlier intervention time-window to account for SAH-induced EBI [5, 75].

Almost one-fifth of patients (19%, 27) were excluded from the study due to initial predictions that they were unlikely to survive for more than 48 h after admission. This selection bias may have led to an over-representation of patients with good WFNS grade SAH (60%, 30/50) and therefore a possible ceiling effect for favourable GOSE outcome regardless of whether Cerebrolysin was administered. Observations have been made in other studies whereby the beneficial effects of Cerebrolysin was more pronounced among patients that had poor-grade SAH or more severe pathologies such as ischemic stroke, TBI or Alzheimer’s disease [35, 40, 44, 73, 74, 78]. Since up to 26% of patients with WFNS grade V SAH can experience good longterm mRS (i.e. 0–2) outcomes with standard treatment, we recommend that subsequent studies should consider recruiting more patients with poor-grade SAH [79, 80].

The optimum dose for Cerebrolysin in the setting of aneurysmal SAH requires further investigation. Experimental studies investigating the effect of Cerebrolysin in murine models for focal ischemic stroke noted that a threshold daily dose of 2.5 ml/kg to 5 ml/kg was required before significant reductions in infarct volume or improvements in functional outcome could be observed [20, 26]. Apart from one clinical ischemic stroke trial that concluded a daily 50 ml dose of Cerebrolysin for 10 days resulted in better 30-day NIHSS scores than a lower dose of 10 ml, no other trial has compared different dosing regimens [71]. We adopted a 30 ml daily dose protocol from previous positive ischemic stroke RCTs, but since the minimally-effective weight-based dosages determined from animal studies are considerably higher than that prescribed for human subjects, it is possible that Cerebrolysin group patients may have received inadequate doses [32, 34, 43].

Cognitive dysfunction is an increasingly recognized cause of disability in SAH survivors occuring in 7 to 15% of patients [81, 82]. In view of the positive clinical results of Cerebrolysin in treating vascular dementia and Alzheimer’s disease, we hypothesized that an improvement in neurocognitive performance could be detected in SAH patients [83, 84]. The negative results of this study may have been due to the relatively short two-week course of administration that was designed to predominantly exploit Cerebrolysin’s neuroprotective action. Conversely for dementia RCTs, four- to 12-week Cerebrolysin regimens were selected to principally harness its neurorestorative capacity [78, 83]. Therefore in designing SAH trials assessing neurocognition, longer Cerebrolysin exposure durations, for example at least 4 weeks to incorporate the post-SAH recovery phase, should be considered.

The NIH/ NINDS CDE Project initiative advised that a broader range of outcome instruments be utilised for SAH trials since no single grading scale was considered sufficiently accurate [56]. This study addressed this by performing multi-dimensional outcome assessments in order to establish a framework for more focused evaluations in a future phase III trial. We propose that global functional outcomes continue to be assessed by six-month GOSE and mRS. From our findings of six-month favourable GOSE outcomes in each study group (76% for Cerebrolysin versus 68% for saline), a future phase III clinical trial with an alpha level of 0.05 and a power of 80%, will require a sample size of 986 patients (i.e. 493 patients per arm). If six-month favourable mRS is designated as the primary endpoint (88% for Cerebrolysin versus 68% for saline from the present study), a considerably more feasible sample size of 132 (i.e. 66 per arm) will be necessary. We propose that other outcome measures such as MOCA, DCI and QoL, should continue to be assessed as secondary study endpoints. Otherwise adequately powered trials to primarily detect significant differences in these outcomes between groups would require recruiting more than 4500 subjects in total.

## Conclusions

The findings of this pilot trial support the safety and feasibility of administering Cerebrolysin to aneurysmal SAH patients. No significant benefit for global functional outcomes and neurocognitive performance was observed. An earlier intervention time-window, a longer duration of drug adminstration, a larger trial to assess six-month GOSE or mRS, along with the recruitment of a more homogenous cohort of moderate to severe WFNS grade SAH patients may impart greater insight into Cerebrolysin’s potential therapeutic role.

## Data Availability

The datasets acquired and analyzed for this study are available from the corresponding author upon reasonable request.

## Abbreviations

AHA: American Heart Association
APACHE: Acute Physiology and Chronic Health Evaluation
ASA: American Stroke Association
BI: Barthel Index
CCI: Charlson Comorbidity Index
CI: Confidence interval
CONSORT: Consolidated Standards of Reporting Trials
CT: Computed tomography
DCI: Delayed cerebral ischemia
EBI: Early brain injury
EEG: Electroencephalography
GCS: Glasgow Coma Scale
GOSE: Glasgow Outcome Scale Extended
IL: Interleukin
MCA: Middle cerebral artery
mRS: Modified Rankin Score
MOCA: Montreal Cognitive Assessment
NCSE: Neurobehavioural Cognitive State Examination
NIH: National Institutes of Health
NIHSS: National Institutes of Health Stroke Scale
NINDS: National Institute of Neurological Disorders and Stroke
OR: Odds ratio
QoL: Quality of Life
RCT: Randomized controlled trial
SAE: Severe adverse effect
SAH: Subarachnoid hemorrhage
SF-36: 36-Iten Short Form Health Survey questionnaire
SS-QOL: Stroke-specific QOL Scale
TBI: Traumatic brain injury
TNF: Tumor necrosis factor
WFNS: World Federation of Neurosurgical Societies
